# Supplementary material for: Protocol for a scoping review investigating success in research capacity building for nurses, midwives and allied health professionals
Source: PLoS One. 2025 Aug 1;20(8):e0329264. doi: 10.1371/journal.pone.0329264 (PMC12316307; doi:10.1371/journal.pone.0329264)
Supplement: S2 Appendix — (DOCX) [file pone.0329264.s002.docx]

| **Author** | **Title** | **Year of Publication** | **Participants** | **Context** | **Study Methods** | **Descriptions of aims or success** |
| --- | --- | --- | --- | --- | --- | --- |
|  |  |  |  |  |  |  |
|  |  |  |  |  |  |  |
|  |  |  |  |  |  |  |
|  |  |  |  |  |  |  |
|  |  |  |  |  |  |  |
|  |  |  |  |  |  |  |
|  |  |  |  |  |  |  |
|  |  |  |  |  |  |  |
|  |  |  |  |  |  |  |
|  |  |  |  |  |  |  |
|  |  |  |  |  |  |  |
|  |  |  |  |  |  |  |
|  |  |  |  |  |  |  |
|  |  |  |  |  |  |  |
|  |  |  |  |  |  |  |
|  |  |  |  |  |  |  |
|  |  |  |  |  |  |  |
|  |  |  |  |  |  |  |
|  |  |  |  |  |  |  |
|  |  |  |  |  |  |  |
|  |  |  |  |  |  |  |
